# Nursing in Diseases of the Eye, Ear, Nose, and Throat

**Published:** 1910-11

**Authors:** 


**Nursing in Diseases of the Eye, Ear, Nose, and Throat.** By the **Committee on Nurses of the Manhattan Eye, Ear, and Throat Hospital,** New York City. 12 mo volume of 281 pages. Illustrated. Philadelphia and London: W. B. Saunders Company. 1910. (Cloth, $1.50 net.)

This is a practical book prepared by the surgeons who make up the Committee on Nurses at the Manhattan Eye, Ear, and Throat Hospital, New York. The manual is intended for nurses and not for physicians. It contains practically all a nurse needs to know relative to the subject of which it treats and does not go beyond the capacity of well-educated nurses. The illustrations are excellent and arrangement of the work is good. J. A. R.

